# Evaluation of (GTG) 5-PCR for Genotyping of *Klebsiella pneumonia* Strains Isolated from Patients with Urinary Tract Infections

**Published:** 2019-10

**Authors:** Reza RANJBAR, Davoud AFSHAR

**Affiliations:** 1. Molecular Biology Research Center, Systems Biology and Poisonings Institute, Baqiyatallah University of Medical Sciences, Tehran, Iran; 2. Department of Microbiology and Virology, School of Medicine, Zanjan University of Medical Sciences, Zanjan, Iran

**Keywords:** *Klebsiella pneumoniae*, Molecular typing, (GTG) 5-PCR assay

## Abstract

**Background:**

*Klebsiella pneumonia* is one of the common causes of pneumonia and bacteremia in intensive care patients. The present study was aimed to determine the capability of (GTG) 5-PCR assay for molecular typing of *K. pneumonia* strains isolated from patients with urinary tract infections.

**Methods:**

In this descriptive-sectional study, *K. pneumoniae* strains were collected from hospitalized patients with urinary tract infection in Baqiyatallah Hospital, Tehran, Iran during 2017–2018. Isolates were identified by conventional microbiological tests. Bacterial DNA was extracted using boiling method and (GTG) 5-PCR assay was used for subtyping of the isolates. For clustering of isolates, dendrogram was generated according to the un-weighted pair group method with arithmetic (UPGMA).

**Results:**

Overall, 88 *K. pneumoniae* isolates were isolated and subjected to the molecular typing study. The (GTG) 5–PCR assay was able to differentiate the *K. pneumoniae* strains into 9 clusters including G1–G9. Genotype clusters G4 and G9 consist of highest (26) and lowest (1) number isolate, respectively.

**Conclusion:**

The *K. pneumonia* strains isolated under the study belonged to various clones and the (GTG) 5-PCR assay as simple and rapid method can be a powerful tool for molecular typing of *K. pneumoniae* strains.

## Introduction

*Klebsiella pneumoniae* belongs to enterobacteriaceae family, is a Gram-negative nosocomial pathogen which leads to many infections including pneumonia, bacteremia and urinary tract infections usually in the patients with underlying disease such as diabetes mellitus (1, 2). The organism is important because of its ability to produce many enzymes which destroy extended spectrum of antimicrobial agents. From these, *K. pneumoniae* carbapenemases (KPCs) are important β-lactamases and hydrolyze cephalosporins, penicillin, carbapenems and monobactams (3, 4). Infections due to KPCs- positive *K. pneumoniae* have a high mortality rate in compare with those caused by carbapenems susceptible isolates (5, 6). Other β-lactamases are also produced by species including OXA, TEM, SHV and CTX-M (7, 8). The treatment of *K. pneumoniae* infections are more complicated and in the many cases, it will be ineffective (9).

The different genotypes of *K. pneumoniae* have different susceptibility or resistance profiles to antimicrobial agents (10). Besides, finding the source of nosocomial infections resulted from *K. pneumoniae* can be helpful in management of outbreaks. Molecular genotyping is a useful approach for identifying probable sources of infections by determining bacterial genetic profiles (11). Moreover, identification of bacteria with genotypic methods lead to better identifying some features of bacteria, including increased virulence and transmissibility (12).

Several genotyping methods have been introduced from a long time ago, totally classified into two groups based on PCR and enzymatic digestion methods. The PCR based assays are also depending on the sequencing or randomly amplified patterns analysis. Random PCRs methods are simple and cost-effective and easily can apply to many microorganisms (13).

(GTG) 5-PCR fingerprint approach is a novel genotyping method and performs with a single poly-trinucleotide (GTG) primer targeting a conserved and repetitive poly GTG sequences present in bacterial genomes. The assay effectiveness has been deemed acceptable in compare with other molecular typing analysis (14).

We aimed to determine the genetic diversity of *K. pneumoniae* isolates using (GTG) 5-PCR molecular typing technique.

## Materials and Methods

### Bacterial Isolates

The study included all *K. pneumoniae* isolates recovered from urinary tract infections of patients hospitalized in Baqiatallah Hospital, Tehran, Iran during 2017–2018. These isolates were identified by standard biochemical tests.

The study (No.91002360) was approved by the Ethics Committee of Baqiyatallah University of Medical Sciences.

### DNA extraction

DNA extraction carried out using method described previously with some modifications (15).

All isolates were cultured on LB broth (Merk, Germany) and incubated at 37 °C for 24 h. Following overnight incubation, each of bacterial suspensions was centrifuged at 5000 rpm for 5 min and bacterial sediments suspended in 250 μl of lysis buffer (10 mM Tris-HCl, 0.1 M NaCl, 1 mM EDTA, 5% [v/v] Triton X100, pH 8.0). The suspensions were separately boiled at 100 °C for 3 min and centrifuged at 10000 rpm for 10 min. The supernatants were transferred into sterile microtubes and about 250 μl of ethanol 95% added to each microtube, incubated at −20 °C for 30 min. Following centrifugation at 14000 rpm for 20 min, the sediments were dissolved in 100 μl sterile distilled water and stored at −20°C. Quality of DNA was evaluated using gel agarose electrophoresis.

### (GTG) 5-PCR assay and PCR pattern analysis

(GTG) 5-PCR was used for subtyping of the strains. The amplification reaction was carried out in a total volume of 25 μl containing 1.0 U of Taq DNA polymerase (Dongsheng Biotech, Guangzhou, China), 1.0 nM of primer (5'GTGGTGGTGGTGGTG3'), 2.5 mM MgCl2, 0.25 mM of each dNTP, and 40 ng of genomic DNA. PCR conditions were as follows: initial denaturation at 94 °C for 3 min; 30 cycles of denaturation at 94 °C for 60 s, annealing at 52 °C for 60 s, and extension at 72 °C for 5 min; followed by a final extension at 72 °C for 7 min. PCR amplicons were electrophoresed on gel aga-rose 1%, stained with DNA safe stain (Cinaclone, Iran) and finally visualized under UV light transilluminator (Fotodyne, USA).

Genetic relationships among *K. pneumoniae* isolates were analyzed using GelCompar II version 5.10 with unweighted pair group method with arithmetic (UPGMA) clustering method.

## Results

**Fig. 1:**
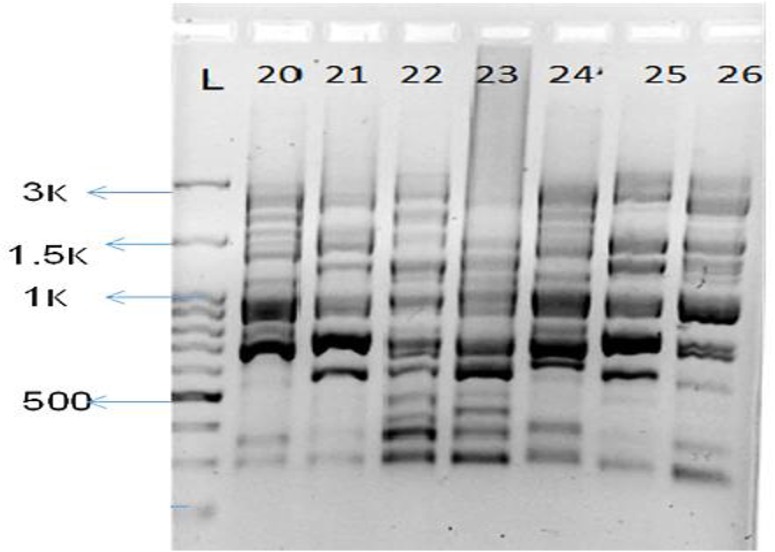
The (GTG) PCR fingerprints of several *K. pneumoniae* isolates (20–26)

Overall, 88 *K. pneumoniae* isolates were isolated from patients' specimens and subjected to genotypic analysis. The PCR with the (GTG) 5 oligonucleotide yielded the fingerprints with band numbers ranging from 15 to 20, approximately (Fig. 1). An amplicon with size of 900bp was amplified in the all of isolates, while amplicons with size of 900 and 1000bp just observed in some isolates.

**Fig. 2:**
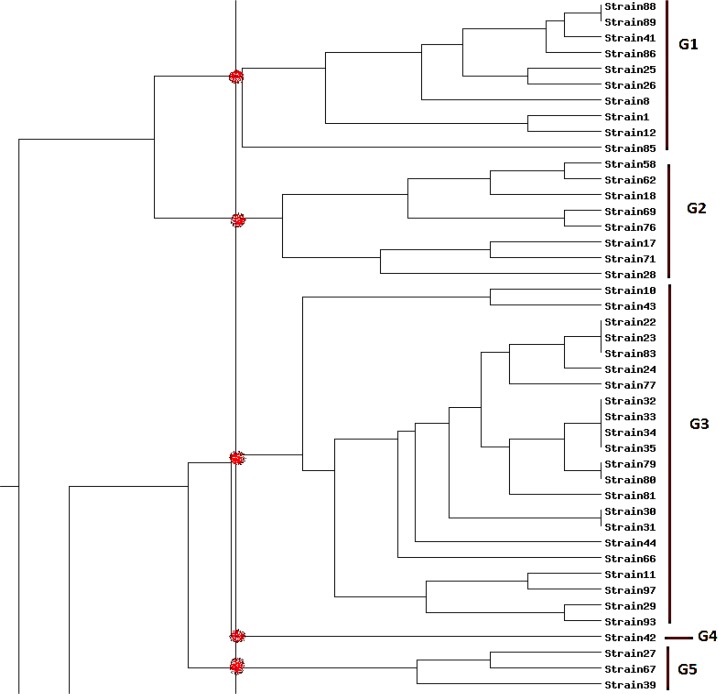

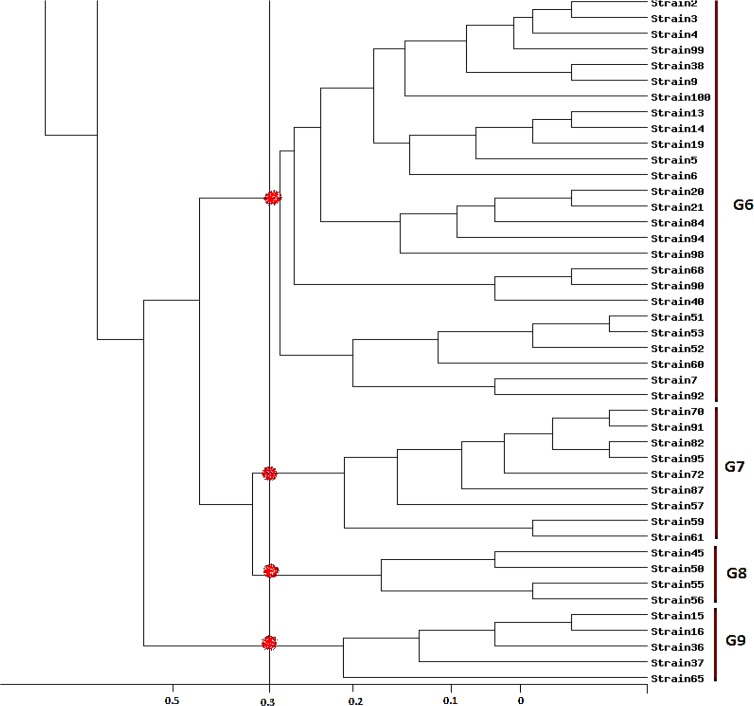
Dendrogram based on (GTG)5-PCR fingerprints derived from 19 *K. pneumoniae* clinical isolates. Comparison method: Dice Clustering method: UPGMA

All of isolates were successfully genotyped by (GTG) 5-PCR and classified in 9 clusters (G1–G9) with similarity level of 70% (Fig.2). The number of isolates in each genotype were as follow; G1(10), G2(8), G3(22), G4(1), G5(3), G6(26), G7(9), G8(4) and G9(5).

## Discussion

*Klebsiella* genus is one of the most important Gram-negative opportunistic bacterium that causes severe diseases such as septicemia, pneumonia and urinary tract infections (16). Because of the increasing of the antibiotic-resistant *Klebsiella* in the hospital environments, the identification of contamination sources is important (17). Biochemical typing, such as the AP-20E, is ineffective in clinical microbiology laboratories due to the similarities of several species biochemical profiles (18–20). In recent decade, molecular typing is increasing in microbiology laboratories. From many molecular typing methods for bacterial fingerprinting, PCR based assays are easy and cost-effective methods with reliable discrimination. These assays require just a low amount of DNA which may be so low in some conditions such as DNA extraction from biopsy specimens. Some experiments have been done represented similar results. (GTG) 5-PCR fingerprinting is a rapid and effective method to identification of bacteria.

In compared to other assays, the (GTG) 5-PCR analysis appears to be a hopeful molecular typing method and it has so ability to differentiate many strains within *K. pneumoniae* species (14). Reproducibility of (GTG)5-PCR assay seems to be reliable in compare with other rep-PCR fingerprinting (16). The (GTG)5-PCR was able to characterize all strains belonging to each enterococcal species into separated clusters, successfully (17).

This fingerprinting method has already been applied effectively in genotyping of several bacterial species (18). In another study, (GTG)5-PCR fingerprinting was found to be a rapid and reproducible genotypic method for genotyping of lactobacilli and *Streptococcus mutans* (21, 22). This method can be useful to determine the taxonomic and phylogenetic relationship among acetic acid bacteria (23).

In the (GTG) 5-PCR assay, the number of bands in fingerprints is usually between 8 and 15 and can be variable in different bacterial species (20). However, in our experiment the number of bands in the fingerprints obtained to be 15–20. The (GTG) 5-PCR assay produce band numbers from 10 to 25 and in comparison with other molecular typing methods such as ERIC-PCR, ERIC2-PCR, BOX-PCR and REP-PCR methods, it had a highest Simpson discrimination index (93.68%) (19). There are several factors that can be affect the fingerprints band numbers such as agarose percentage in gel electrophoresis system so that an electrophoretic system with high percent of agarose can better separate and visualize more amplicons (24).

In comparison with lactic bacteria, the number of bands in (GTG) 5-PCR fingerprints is low in the staphylococci. The low number bands in fingerprints can be discriminative in small genomic variations and can be considered as advantage for GTG) 5-PCR assay (25).

## Conclusion

The (GTG) 5 -PCR analysis provides a promising tool for molecular typing of *K. pneumoniae* isolates.

## Ethical considerations

Ethical issues (Including plagiarism, informed consent, misconduct, data fabrication and/or falsification, double publication and/or submission, redundancy, etc.) have been completely observed by the authors.
